# Applications of Capillary Electrophoresis for the Detection of Adulterants in Dietary Supplements

**DOI:** 10.3390/ph17091119

**Published:** 2024-08-24

**Authors:** Gabriel Hancu, Blanka Székely-Szentmiklósi, Denisa Gabriela Stroia, Hajnal Kelemen

**Affiliations:** Department of Pharmaceutical Chemistry, Faculty of Pharmacy, “George Emil Palade” University of Medicine, Pharmacy, Science and Technology of Târgu Mureș, 540142 Târgu Mureș, Romania; blanka.szekely-szentmiklosi@umfst.ro (B.S.-S.); stroia471@gmail.com (D.G.S.); hajnal.kelemen@umfst.ro (H.K.)

**Keywords:** dietary supplements, capillary electrophoresis, adulteration, quality control, natural products

## Abstract

In recent years, the consumption of dietary supplements, particularly those incorporating plant-based ingredients, has increased greatly, driven by the perception of their natural origins and purported minimal health risks. However, one significant safety concern revolves around the adulteration of dietary supplements, wherein unscrupulous manufacturers may illegally incorporate pharmaceutical substances or their analogs into these products to achieve increased efficiency and bolster sales. This review assesses the role of capillary electrophoresis (CE) in ensuring the quality control of dietary supplement products over the past two decades. This study provides an overview of various applications of CE in analyzing dietary supplements, outlining the typical attributes of natural product analysis using CE. These analyses demonstrate the broad versatility of CE, exemplified by its diverse applications and detection modes. Moreover, the review highlights the growing prominence of CE as a separation technique in quality control, by comparison with more conventional methods like high-performance liquid chromatography (HPLC). Through this exploration, the review elucidates the pivotal role of CE in upholding the integrity and safety of dietary supplements, in connection with a landscape of evolving regulatory challenges and consumer demands.

## 1. Introduction

A dietary supplement, also known as a food supplement, is designed to complement the diet by providing essential nutrients or other substances that may be lacking in the regular food intake. These supplements typically contain various dietary ingredients such as vitamins, minerals, herbs, amino acids, or other substances. They are available in diverse pharmaceutical formulations, including capsules, pills, tablets, liquids, powders, or energy bars. Dietary supplements are not intended to replace food but rather to enhance nutritional intake, addressing deficiencies resulting from inadequate diet, lack of physical exercise, or unhealthy life habits. People use dietary supplements for a wide range of purposes, including achieving dietary balance, maintaining overall health, preventing chronic illnesses, enhancing physical appearance, promoting mental well-being, improving sexual performance, or boosting sports performance, among others. One key reason for their popularity is the perception that they are safer than synthetic drugs, as they can be obtained without a medical prescription and are readily available in pharmacies, retail outlets, and online platforms. While dietary supplements are regulated differently from drugs and do not require pre-market approval by regulatory agencies like the Food and Drug Administration (FDA) or European Medicines Agency (EMA), they must adhere to specific regulations regarding labeling, safety, and product composition and claims [1,2].

The adulteration of dietary supplements presents a multifaceted challenge for authorities, involving deceptive practices aimed at compromising both consumer confidence and product integrity. Manufacturers engage in covert tactics by incorporating potent pharmaceuticals, such as erectile dysfunction medications (5-phosphodiesterase inhibitors like sildenafil, tadalafil, vardenafil) and anorectic amphetamines (like amfepramone, phentermine, sibutramine), into supplements. Additionally, unscrupulous producers may include banned, unapproved, or hazardous substances to artificially enhance the perceived effectiveness of their products. Moreover, adulteration often involves diluting active ingredients with inferior substitutes as a cost-saving measure, compromising the quality of the product. Insufficient oversight during manufacturing processes heightens the risk, allowing contaminants such as heavy metals or microbial toxins to contaminate supplements, posing significant health risks to consumers. Furthermore, mislabeling and dissemination of false efficiency claims exacerbate the issue, perpetuating misinformation and undermining informed consumer decision-making [3,4].

The primary method of adulteration persists as the inclusion of pharmaceuticals or their analogs in dietary supplements, a practice aimed at enhancing the supplement’s efficacy. This approach not only exacerbates the perceived benefits of the product but also increases consumer confidence in its effectiveness. The presence of approved pharmaceutical analogs (designer drugs), which lack approval for therapeutic use as prescription drugs, presents a notable health risk to consumers due to their unexplored efficacy and potential toxic effects, and can result in unforeseen adverse outcomes. Adulterated products often exhibit a spectrum of adulteration, ranging from instances where a single drug is detected (common) to rare occurrences where multiple distinct drug ingredients can be identified. Compounding this issue is the deceptive marketing strategy employed by many manufacturers, who promote their products as “natural,” leading consumers to perceive them as inherently safer and healthier alternatives to conventional synthetic pharmaceuticals [5].

Dietary supplements commonly targeted for adulteration often belong to categories addressing erectile dysfunction, weight loss, and bodybuilding or athletic performance enhancement. These products, susceptible to adulteration, are frequently marketed with promises of rapid results and improved physical performance. Moreover, dietary supplements have proliferated in the global market, easily accessible to consumers through various channels including supermarkets, drugstores, natural health/food stores, herbal shops, or gyms. They are also readily available for purchase via television sales and online platforms utilizing the Internet. Over the past decade, there has been a significant increase in the distribution of dietary supplements originating from the illegal market, further compounding the issue [6].

**Figure 1:**
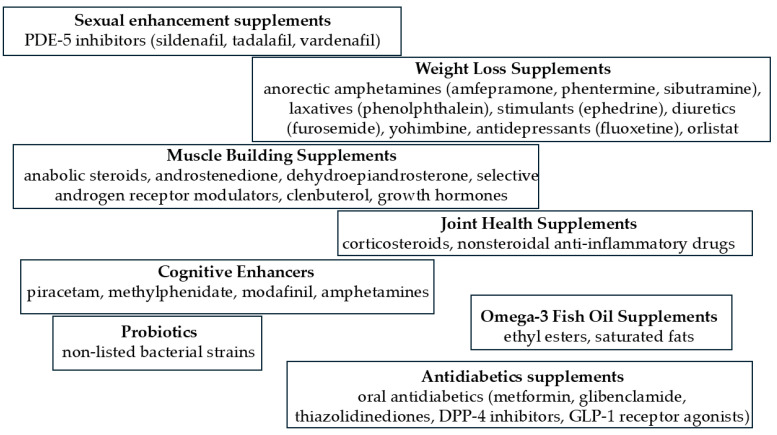
Most adulterated dietary supplements—prevalence of adulteration in dietary supplements by categories [4,5,6,7].

Figure 1 presents the most adulterated types of dietary supplements and examples of adulterants most frequently used in their falsification.

The adulteration of dietary supplements is influenced by a multifaceted array of motives, ranging from financial gain to meeting consumer demands for swift and potent solutions. Given the significant economic stakes in the global trade of dietary supplements, with consumers in the USA alone spending over USD 20 billion annually on these products, they are highly susceptible to adulteration driven by economic incentives aimed at maximizing profits. Unethical manufacturers may strategically incorporate pharmaceutical substances or their analogs to not only enhance product efficacy but also gain a competitive advantage in the market, all while evading regulatory scrutiny [7].

The selection of sample preparation methods and analytical techniques for detecting, identifying, and quantifying target substances in adulterated dietary supplements is contingent upon several factors. These factors encompass the quantity and diversity of compounds being targeted, the desired sensitivity level, the formulation type, and the complexity of the sample matrix. For example, samples containing several botanical ingredients may pose additional analytical challenges owing to their complex composition [3].

Various analytical techniques play a crucial role in detecting adulterants in dietary supplements, ensuring compliance with safety regulations. High-performance liquid chromatography (HPLC) is a widely utilized method for separating and quantifying components in complex mixtures, often coupled with ultraviolet (UV) or mass spectrometry (MS) detection for heightened sensitivity. Liquid chromatography–mass spectrometry (LC-MS) combines the separation capabilities of LC with the precise identification and quantification afforded by MS, facilitating accurate analysis of analytes. Gas chromatography–mass spectrometry (GC-MS) excels in detecting volatile and semi-volatile compounds, leveraging distinctive MS patterns for identification. For initial screening, thin-layer chromatography (TLC) can serve as a cost-effective tool, utilizing stationary phase separation principles to detect potential adulterants [8,9].

In addition to these separation techniques, nuclear magnetic resonance (NMR) spectroscopy provides valuable insights into molecular structures, aiding in the precise identification of adulterants based on their distinct chemical signatures. Immunoassays have gained prominence for their rapid and sensitive detection capabilities, utilizing antibodies’ specificity to discern and quantify target analytes in dietary supplements. Lastly, DNA barcoding has emerged as an innovative approach, utilizing genetic sequencing to authenticate plant ingredients and uncover any potential adulterants or substitutions [10,11].

These methods are utilized either individually or in combination to thoroughly analyze dietary supplements, ensuring effective adulterant control and upholding product safety.

## 2. Overview of Capillary Electrophoresis

Capillary electrophoresis (CE) encompasses a spectrum of electromigration techniques that employ narrow capillaries to achieve highly efficient molecule separation, regardless of their size or charge. Its adaptability is underscored by various separation modes, notably capillary zone electrophoresis (CZE) and micellar electrokinetic chromatography (MEKC). This versatility, along with other advantageous features (fast analysis time, low consumption of analytes and reagents, relatively low operational costs), makes CE particularly well-suited for the intricate analysis of natural products. While CZE traditionally focused on separating charged analytes, MEKC has expanded CE’s capabilities to include both charged and neutral molecules, thereby enhancing overall separation efficiency [12].

CZE represents the most straightforward CE technique, relying on the principle of separating analytes based on their charge-to-size ratio in an electric field. CZE capitalizes on the differential migration velocities of analytes, driven by their respective charges and sizes, when subjected to an applied voltage that creates an electric field across the capillary. This phenomenon, known as electrophoretic flow, dictates the movement of ions within the electric field. The pH of the background electrolyte (BGE) plays a crucial role in determining the dissociation states of analytes. Furthermore, non-dissociated and uncharged molecules can migrate through the capillary via electroosmotic flow (EOF), facilitated by the movement of the bulk solution in the capillary [13].

In MEKC, charged surfactants such as sodium dodecyl sulfate (SDS) are introduced into the BGE, resulting in the formation of dynamic micelles. These micelles migrate electrophoretically like other charged species when subjected to an applied voltage. Acting like a stationary phase in chromatography, these micelles are referred to as the “pseudostationary phase” due to their migratory nature, while the surrounding aqueous BGE solution serves as the “mobile phase”. Uncharged analytes may be incorporated into the hydrophobic core of the micelles, leading to separation based on differences in analyte partitioning between the micellar pseudostationary phase and the aqueous buffer solution. Moreover, the presence of charged micelles facilitates ionic interactions, thereby altering the CE separation selectivity of charged solutes [14].

To ensure effective CE analysis, the following conditions should be considered: BGE composition, BGE concentration, BGE pH, BGE additives, voltage, temperature and injection parameters [12,13,14]. The optimal BGE composition varies depending on the analytes and matrix under study; for example, a phosphate BGE may be utilized due to its buffering capability and compatibility with various additions. The pH of the BGE is critical in defining the dissociation state of the analytes, which influences their charge and migration behavior; it is critical to adjust the pH to the appropriate level for the analytes [13]. Surfactants or other additives (organic solvents) may be used in the BGE to improve the separation of certain analytes; for example, SDS is frequently employed in MEKC to separate charged and neutral molecules [14]. The applied voltage and temperature are crucial in determining migration speed and separation efficiency; typical circumstances may include a voltage range of 15–30 kV and a temperature range of 20–30 °C, which can be changed based on the analysis’s specific requirements [12,13].

In CE, detection strategies play a pivotal role in analyzing natural products. UV detectors, including fixed and variable wavelength types such as diode array detectors (DADs) and photodiode arrays (PDAs), maintain popularity due to their widespread availability in commercial CE instruments. However, there is a shifting trend towards the use of MS detection, which offers an attractive combination for identifying and confirming components in complex mixtures. The increasing utilization of CE-MS tandem systems (CE-MS/MS) further reinforces its path towards becoming a routine analytical technique [15].

While numerous articles have been published in recent decades focusing on the applications of CE in dietary supplement and food analysis, there remains a notable gap in the literature concerning its utilization specifically for adulterant detection [16,17,18,19,20].

Ganzera (2008) evaluated the importance of CE in strengthening the quality control of herbal medicinal products, especially considering the increasing demand for traditional Chinese and Indian remedies in Europe and the USA; the review underscores the role of CE as both an alternative and complementary approach to conventional methods such as HPLC [16].

Rabanes et al. (2012) conducted an extensive review of published research and review articles, revealing a notable increase in the utilization of electroseparation techniques, specifically CZE and electrokinetic chromatography (EKC), in the analysis of natural products, during the period from 2006 to 2010. They provided an overview of diverse applications in natural product analysis, including pharmaceuticals/herbal products, fingerprinting, quality control, food contaminants, and toxicological compounds relevant to forensics. Additionally, the review delves into key aspects such as detection strategies, microchips, sample preconcentration, and chiral separations, shedding light on both existing research and unexplored areas. Furthermore, fundamental concepts concerning CZE and EKC, and their suitability for analyzing natural products, are briefly discussed [17].

Gackowski’s (2021) review article synthesizes scientific findings from 2010 to 2019 concerning the application of CE in quantifying active constituents, such as phenolic compounds, coumarins, protoberberines, curcuminoids, iridoid glycosides, alkaloids, and triterpene acids, in medicinal plants and herbal formulations. The examination underscores the prevalence of CE with UV detection as the primary electromigration technique for selectively separating and quantifying bioactive compounds. To enhance the resolution and sensitivity, various detection methods, including MS, are employed alongside BGE modifiers and different extraction and pre-concentration techniques. Ultimately, the review concludes that CE is a potent tool comparable to HPLC for certain applications. Its advantages include rapid method development, high efficiency, versatile separation modes, and minimal solvent and sample consumption, rendering it an attractive and environmentally friendly alternative for drug quality control and raw plant material analysis [18].

Przybylska et al.’s (2021) review article provided a comprehensive summary of scientific reports spanning from 2005 to 2021 concerning the application of CE in the analysis of polyphenolic compounds, coumarins, amino acids, and alkaloids present in teas or various plant parts used for aqueous infusions. The analysis reveals that more than 60% of the articles included originate from China. It highlights that CE with UV detection emerges as the predominant technique for selectively separating polyphenolic and flavonoid compounds. However, the utilization of CE-MS proved promising, enabling sensitive analyte determination with lower limits of detection, and holding potential for routine application in functional food analysis. Furthermore, the integration of modifications in electrochemical techniques not only enhances method sensitivity but also reduces analysis time, representing a significant advancement in the field [19].

Muschietti et al. (2020) published a review with the aim to provide a comprehensive overview, spanning from 2009 to 2019, of the most encountered adulterants in dietary supplements intended for the treatment of conditions like erectile dysfunction, obesity/overweight, diabetes mellitus, and hypertension. The analysis reveals a prevalent occurrence of dietary supplements adulterated with undisclosed synthetic compounds or their unauthorized analogues, posing significant risks to consumers as well as challenges for regulatory agencies. Despite regulatory efforts, the growing consumption and globalization of markets complicate the control of adulterants, leading to the continual emergence of new ones. Hyphenated techniques such as LC-MS/MS or LC-NMR are highlighted as effective means for the rapid and precise detection and identification of adulterants. However, conventional techniques like LC-UV still play a valuable role in detecting undeclared substances. This review only briefly mentions the utilization of CE techniques for adulteration detection [20].

Considering the aspects presented above, this study aims to explore the applications of CE for the detection of adulterants in dietary supplements. To our knowledge, no review article exclusively addressing the detection of adulterants in dietary supplements using CE has been published so far. By examining the effectiveness of CE techniques in identifying and quantifying adulterants, this research seeks to contribute to the advancement of analytical methods for ensuring the safety and integrity of dietary supplements.

## 3. Applications of CE in Detecting Adulterants

The studies in this section are arranged in chronological order to illustrate the progression of research in using CE for detecting adulterants in dietary supplements, showing how these techniques have evolved over time.

Ku et al. (1999) developed a CE method for the determination of six synthetic amphetamine and amphetamine-like anorexics (clobenzorex, diethylpropion, fenfluramine, methamphetamine, phenylpropanolamine, phentermine) as adulterants in traditional Chinese medicines commercialized in Taiwan. The optimal analytical conditions consisted of a 120 mM phosphate BGE at pH 2.0 with 15% acetonitrile as organic additive, an applied voltage of 16 kV and a temperature of 30 °C. A one-factor-at-a-time (OFAT) strategy was applied for method development. Fluoren-2,7-diammonium chloride was used as internal standard (IS), with detection set at 200 nm. The recovery of anorexic adulterants was investigated using C_8_-SCX mixed solid phase extraction (SPE). Additionally, traditional Chinese medicinal powders underwent analysis using the CE method, and their results were corroborated via GC–MS. Among these samples, clobenzorex, diethylpropion, and fenfluramine were successfully detected and quantified [21]. Previously, the same research group had developed an MEKC method to identify clobenzorex hydrochloride and diazepam adulterants present in traditional Chinese medicine [22].

A comprehensive analysis of sixteen synthetic chemicals (acetaminophen, bucetin, caffeine, diazepam, ethoxybenzamide, fenbufen, flufenamic acid, indomethacin, ketoprofen, mefenamic acid, niflumic acid, oxyphenbutazone, phenylbutazone, prednisolone, salicylamide, sulindac) commonly present in adulterated Chinese medicines was conducted by Cheng et al. (2001) using CE with UV detection and electrospray ionization mass spectrometry (CE-ESI-MS). Their study revealed that while only nine peaks were distinguishable with CE-UV, the application of online CE-MS facilitated clear identification of most compounds. In a real sample of Chinese medicinal preparations, some adulterants were identified based on their migration times and protonated molecular ions. For compounds that co-eluted, more reliable identification was achieved through CE coupled with tandem mass spectrometry (CE-MS/MS) in selected reaction monitoring mode. MEKC utilizing SDS yielded superior separation compared to CZE, enabling the detection of fourteen peaks under optimal conditions with UV detection. They also observed that SDS interference was less pronounced in positive ion mode than in negative ion mode when using MEKC-ESI-MS. Direct coupling of MEKC with ESI-MS allowed for the use of up to 20 mM of SDS if the MS operated in positive ion mode. Thanks to enhanced resolution in MEKC, adulterants could be identified without the necessity of MS/MS coupling [23]. The study highlights the effectiveness of CE-MS for the comprehensive analysis of synthetic chemical drugs in adulterated supplements and underscores the importance of advanced analytical techniques in ensuring the safety and authenticity of traditional medicinal products.

Ku et al. (2003) developed a CE method aimed at simultaneously detecting four oral antidiabetic drugs, acetohexamide, chlorpropamide, glibenclamide, and tolbutamide (sulphonylurea derivatives), used as adulterants in dietary supplements for diabetes management. The optimal electrophoretic conditions involved utilizing a 100 mM phosphate–borate BGE at pH 7.5, with an applied voltage of 15 kV and a temperature of 30 °C. For quantification, 2-(4-hydroxyphenyl) ethyl ammonium chloride was employed as an IS, with detection set at 200 nm. The study examined the effects of BGE concentration, BGE pH, and applied voltage on the separation process using an OFAT optimization strategy. The recoveries of the synthetic drug adulterants within traditional Chinese medicinal formulas ranged from 81.3% to 105.5%. Furthermore, glibenclamide was successfully detected and quantified in a genuine sample of traditional Chinese medicine [24].

**Figure 2:**
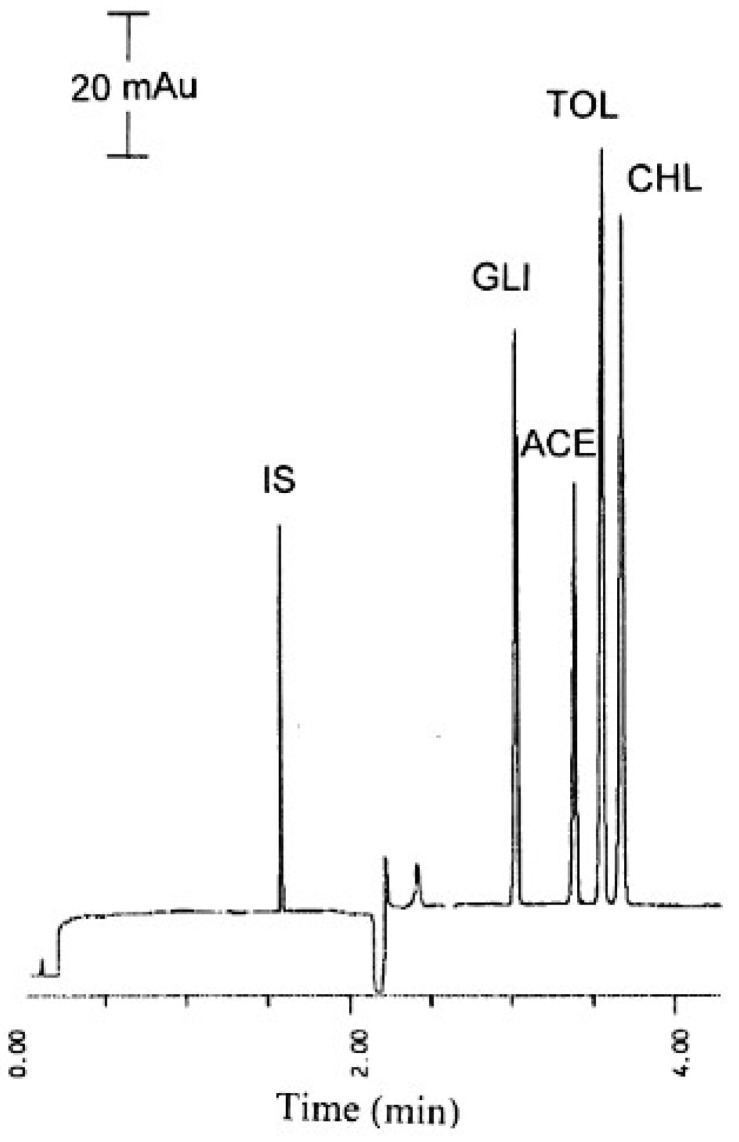
Capillary electropherogram of a mixture of the four synthetic antidiabetic drugs (ACE—acetohexamide; CHL—chlorpropamide; GLI—glibenclamide; TOL—tolbutamide. IS—3-benzyl-5-(2-hydroxyethyl)-4-methylthiazolium chloride) (CE conditions: 100 mM of phosphate, pH 7.5, 15 kV, 30 °C, UV detection 200 nm). Reprinted from Ku et al. [24] with permission from Elsevier.

Figure 2 depicts a CE electropherogram illustrating a mixture of four synthetic anti-diabetic drugs: acetohexamide, chlorpropamide, glibenclamide, and tolbutamide, along with 3-benzyl-5-(2-hydroxyethyl)-4-methylthiazolium chloride used as an IS [24].

Avula and Khan (2004) developed a CE method tailored for the chiral separation of ephedrine alkaloids, which holds significance due to the distinct pharmacological activities exhibited by the different enantiomers. *Ephedra* alkaloids and their homologous compounds, such as (+)-ephedrine, (−)-ephedrine, (+)-pseudoephedrine, (−)-pseudoephedrine, (+)-methylephedrine, (−)-methylephedrine, (+)-methylpseudoephedrine, and norpseudoephedrine, constitute the primary bioactive constituents of *Ephedra sinica* (Ma-Huang), an herb commonly utilized in weight loss and energy-boosting dietary supplements. The study examined various parameters affecting the resolution between the (+) and (−)-enantiomers, including the concentration and pH of the BGE, cyclodextrin (CD) concentration, organic modifier, temperature, and capillary dimensions. It was found that the combinations of β-CD with HP-β-CD as chiral selectors (CSs) had a beneficial effect on the chiral resolution of the peaks. Organic modifiers, particularly methanol, significantly influenced the separation of ephedrine alkaloids, with methanol emerging as the optimal choice when used in phosphate–borate BGE. The developed method was successfully applied to the quantitation of ephedrine alkaloids in dietary supplements. Among the alkaloids commonly found in *Ephedra* extracts, (−)-ephedrine and (+)-pseudoephedrine were identified as the most abundant, with quantities ranging from 0.38% to 1.21% and 0.07% to 0.51% per capsule or tablet weight, respectively [25].

Sitton et al. (2004) conducted a CE study focusing on devising a quantitative method for assessing lipoic acid levels in dietary supplement preparations. Alpha-lipoic acid (thioctic acid) is a compound naturally produced by the body, which plays a crucial role in energy metabolism and acts as an antioxidant, helping to neutralize harmful free radicals. A 50 mM phosphate BGE with 20% methanol (*v*/*v*) at pH 7.0 was used; the analysis required less than 9 min. Limit of detection (LOD) and quantification (LOQ) values of 0.8 and 2.5 μg/mL were achieved [26].

In a study conducted by Sombra et al. (2005), phytopharmaceuticals containing guarana extracts were subjected to analysis using CE, with results compared to those obtained through HPLC. Guarana (*Paullinia cupana Mart.*), a native plant of South America, is primarily valued for its seeds, which are suitable for human consumption; these seeds are characterized by high caffeine concentrations, typically ranging from 3% to 6% (dry weight), along with trace amounts of theophylline and theobromine, and significant quantities of tannins. To establish suitable fingerprints and identify potential adulterants, caffeine was chosen as the marker compound. The main objective of the study was to develop a CE method for quantifying constituents in guarana products, alongside the validation of this method and comparison with HPLC. The study included assessments of elution order, UV spectra, method sensitivity, and precision between HPLC and CE methods. This separation technique was applied to analyze both guarana seed powder and commercial tablets containing guarana. The method’s performance was evaluated in terms of specificity, sensitivity, and precision, with results consistent with those obtained via HPLC. Caffeine levels in the analyzed samples fell within normal concentrations, as expected for non-adulterated products, with neither theophylline nor theobromine detected in the samples under investigation. Moreover, the CE method boasted an analysis time up to two times shorter than HPLC, with minimal solvent consumption [27]. The findings highlight the versatility and efficiency of CE as an analytical tool for quality control and authentication in herbal product analysis.

Phinney et al. (2005) developed three complementary CE techniques aimed at separating and quantifying stereoisomers of ephedrine and pseudoephedrine, including (−)-ephedrine, (+)-pseudoephedrine, (−)-N-methylephedrine, (+)-N-methylpseudoephedrine, (−)-norephedrine, and (+)-norpseudoephedrine. Ephedrine and pseudoephedrine are notable as the primary alkaloids commonly found in *Ephedra sinica*, comprising over 80% of the alkaloid composition within the dried plant material. The detection of ephedrine alkaloids in dietary supplements is crucial for verifying product label claims, especially in products labeled as “ephedra-free”. These methods utilized single or dual CDs as CS systems, enabling enantioselective separation of the target compounds. The researchers applied these methods to analyze five standard reference materials containing *Ephedra* extracts. The results for (−)-ephedrine ranged from 0.31 to 76.43 mg/g, while those for (+)-pseudoephedrine ranged from 0.049 to 9.23 mg/g. The results from the three CE methods were consistent with each other and with results obtained from alternative analytical methods. To confirm the enantiomeric identity of the analytes, known quantities of specific enantiomers were added. The utilization of complementary CE methods enhanced confidence in peak identity, suggesting potential applications of this approach to address other analytical challenges [28].

**Figure 3:**
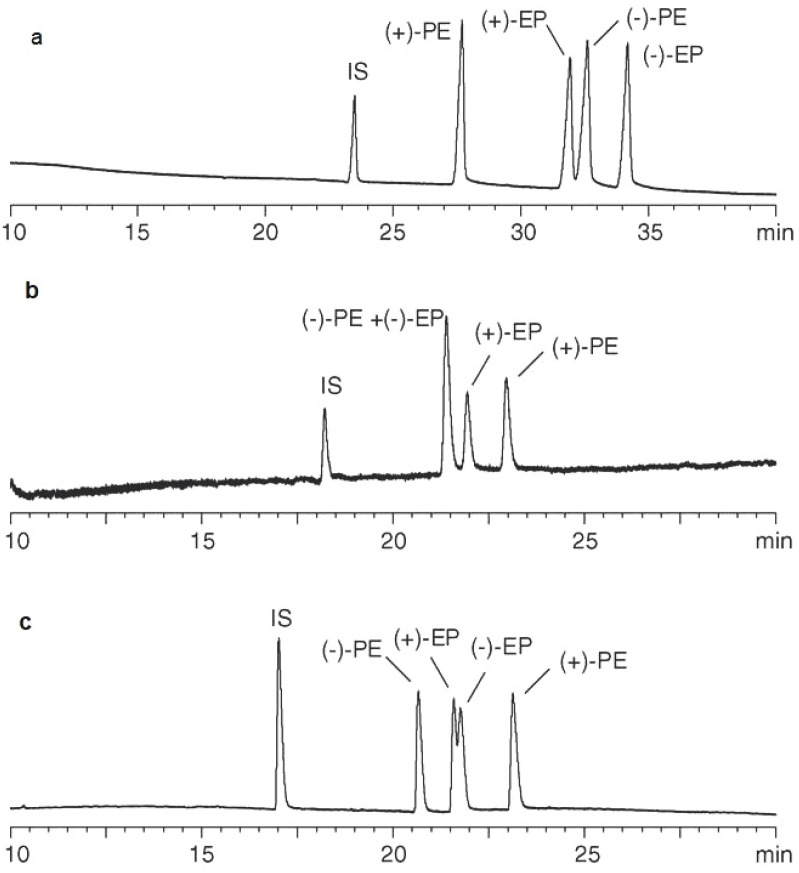
Chiral separation of racemic mixtures of ephedrine and pseudoephedrine with three CS systems (EP—ephedrine, PE—pseudoephedrine) (CE conditions: method (**a**) 5 mM of phosphate, pH 2.5, 2.8% sulfated β-CD + 1.2% heptakis(2,6-di-O-methyl)-β-CD, −15 kV, 25 °C; method (**b**) 25 mM phosphate, pH 2.5, 4% heptakis(2,6-di-O-methyl)-β-CD, 30 kV, 25 °C; method (**c**) 25 mM of phosphate, pH 2.5, 4% hydroxypropyl-β-CD, 25 kV, 25 °C, UV detection 200 nm). Reprinted from Phinney et al. [28] with permission from Elsevier.

Figure 3 illustrates the separation of the stereoisomers of ephedrine and pseudoephedrine along with the IS, α-phenylethylamine, utilizing three CS additive (single and dual) CD systems [28].

Prokorátová et al. (2005) developed three CE methods, including one capillary isotachophoresis (cITP) and two CZE with direct and/or indirect UV detection, for the quantification of *L*-carnitine in various samples. *L*-carnitine is a naturally occurring amino acid found in the human body, primarily in the muscles and liver, which facilitates the transportation of fatty acids to mitochondria, aiding in the conversion of fats into energy. This supports weight reduction, enhances immediate physical performance, increases resistance to physical effort, and protects the heart from strain. The results obtained with the electrophoretic methods were compared with those from a validated HPLC method, yielding comparable outcomes. The LOD of the electrophoretic methods ranged from 2.4 to 4.7 μg/mL. Sample preparation for cITP and CZE using a quinine buffer with indirect photometric detection was straightforward. However, CZE with direct UV detection, which requires derivatization, may not be suitable for routine analysis in industrial laboratories due to its analytical complexity [29].

Dietary supplements containing green tea extracts are increasingly popular due to their catechin content, like (−)-epigallocatechin, (+)-catechin, (−)-epigallocatechin-3-gallate, (−)-epicatechin, or (−)-epicatechin gallate. Catechins, derived from green tea, polyphenolic compounds believed to possess antioxidant properties, act as free radical scavengers and exhibit potential chemo-preventative effects, including benefits against coronary heart disease and the regulation of high blood pressure. In a study conducted by Weiss et al. (2006), the quantification of five catechins in green tea extract dietary supplements was achieved through a methanol:water (4:1) extraction process followed by MEKC with UV detection. The study utilized an optimal BGE comprising 5 mM of borate and 60 mM of phosphate with 50 mM of SDS at pH 7.00. The method had an LOD ranging from 2 to 3 μg/mL and an LOQ ranging from 6 to 8 μg/mL. While the expected catechin compounds were identified in the green tea extracts, the analysis also revealed the presence of unknown compounds, some of which were not polyphenolic in nature. This suggests the possibility of adulteration or contamination in the dietary supplements. Moreover, significant variability was observed in the catechin content among different capsules and batches of the same manufacturer’s product, highlighting potential inconsistencies in product quality and formulation [30]. The study underscores the importance of rigorous quality control measures in green tea extract dietary supplements to ensure the accuracy and consistency of catechin content; the presence of unknown compounds warrants further investigation to ensure product safety and efficacy.

Cianchino et al. (2008) analyzed four distinct phytopharmaceutical dosage forms used for weight loss, including two ground herbal blends and their corresponding infusions, as well as a capsule and a tincture. The aim was to detect potential adulterants commonly found in such products, which could pose unpredictable health risks to consumers. The investigated compounds, including caffeine (a stimulant), furosemide (a diuretic), norephedrine, and ephedrine (both stimulants with decongestant and anorexigenic properties), were aligned with the purported pharmacological effects of the analyzed remedies. The researchers developed and validated a CZE method for the efficient determination of active compounds within a short analysis time, less than seven minutes. The optimized experimental conditions included using 20 mM borate BGE at pH 9.2, a voltage of 30 kV, and a temperature of 25 °C. The LOD and LOQ values were determined as follows: 0.42 µg/mL and 1.40 µg/mL for ephedrine, 0.47 µg/mL and 1.40 µg/mL for norephedrine, 0.12 µg/mL and 0.48 µg/mL for caffeine, and 0.22 µg/mL and 0.73 µg/mL for furosemide. Importantly, the presence of common constituents in the samples did not interfere with the detection of potential adulterants [31].

Malavaki et al. (2008) developed a CE method for quantifying chondroitin sulfate concentrations and screening for impurities such as other glycosaminoglycans, as well as determining hyaluronan impurities in various formulations including raw materials, tablets, hard capsules, and liquid formulations. Chondroitin sulfate, commonly utilized in osteoarthritis treatment, is covalently linked to proteins, and is primarily found in the extracellular matrix of connective tissues. The CE method, employing reversed polarity and a low pH phosphate buffer, demonstrated high sensitivity, with a lower limit of quantitation (LLOQ) of 30.0 μg/mL for chondroitin sulfate and 5.0 μg/mL for hyaluronan. Analysis of eleven commercially available products revealed the presence of hyaluronan impurities in most of them, reaching up to 1.5%. Additionally, CE analysis following treatment with chondroitinase ABC and ACII confirmed the presence of hyaluronan impurities and revealed significant structural differences in chondroitin sulfate, potentially impacting therapeutic outcomes. This developed methodology allows for the direct analysis of chondroitin sulfate products, facilitating quantification, integrity screening, charge density estimation, identification of other negatively charged impurities (such as glycosaminoglycans), and hyaluronan impurity quantification. CE proved valuable for quality control of chondroitin sulfate raw materials and formulations, enabling comprehensive analysis of impurities and structural variations in chondroitin sulfate products, which may influence therapeutic efficacy [32].

Orlandini et al. (2008) developed a CE method for the quality assessment of nutraceuticals containing resveratrol. Resveratrol, a phytoalexin found in various plant sources, is believed to contribute to the cardioprotective effects associated with wine consumption and is advocated as an antioxidant for the prevention of atherosclerosis. The optimization process involved separating eleven components found in effervescent tablets, including active compounds such as vitamin C, riboflavin (vitamin B2), flavanones, and hydroxycinnamic acids. Flufenamic acid was employed as an IS. The response surface methodology was utilized to explore the effects of BGE concentration, acetonitrile percentage, and applied voltage on the determination. This study employed design of experiments (DoE) strategies, which is a notable approach in the development of CE methods for the analysis of dietary supplements. Critical resolution values and analysis time were considered as responses, and the optimal conditions were determined using the Derringer desirability function. The chosen BGE comprised 23 mM of borate buffer adjusted to pH 10.0 with 1 M sodium hydroxide, supplemented with 7% (*v*/*v*) acetonitrile, resulting in an analysis time of less than 7 min. The method’s performance was evaluated according to International Conference on Harmonization (ICH) guidelines, assessing selectivity, robustness, linearity and range, accuracy, precision, and system suitability [33].

Sanchez-Hernandez et al. (2010) conducted a study utilizing a CE-ESI-MS/MS methodology to identify and quantitatively determine the two enantiomers of the non-protein amino acid carnitine (*L*- and *D*-carnitine) in twenty-two dietary food supplements, including drinks, biscuits, capsules, and tablets. Carnitine exhibits distinct biological activities between its enantiomers, while *L*-carnitine is naturally occurring and possesses beneficial pharmacological and nutritional properties—acting as an endogenous cofactor to enhance carbohydrate metabolism, reduce intracellular accumulation of toxic metabolites under ischemic conditions, and aid in the transport of long-chain fatty acids. *D*-carnitine has been found to act as a competitive inhibitor of active uptake systems for *L*-carnitine and can be considered a chiral impurity. The MS/MS experiments were optimized to attain the requisite high sensitivity and selectivity for food analysis. The LOD was approximately 10 ng/mL, enabling the detection of enantiomeric impurities (*D*-carnitine) at levels up to 0.025% of carnitine in foods. The results revealed an *L*-carnitine content ranging from 47% to 115% with respect to the labeled content. Notably, *L*-carnitine was detected in twenty-one out of the twenty-two samples analyzed, with its enantiomeric impurity (*D*-carnitine) detected at levels up to 6%. Furthermore, the presence of racemic carnitine (not permitted by legislation) in one of the twenty-two samples was confirmed, underscoring the method’s potential in ensuring the quality and safety of foods containing carnitine [34].

In a study developed by de Carvalho et al. (2012), a CZE method coupled with capacitively coupled contactless conductivity detection (C^4^D) was developed for the simultaneous determination of amfepramone, fenproporex, sibutramine (anorexics), and fluoxetine (a selective serotonin reuptake inhibitor (SSRI) antidepressant). The researchers utilized a home-made CE system in their experiments. The optimized conditions for the CZE separation were established as follows: 50 mM of phosphate buffer at pH 5.0 in a 50/50 (*v*/*v*) mixture of water/acetonitrile as BGE, 15 kV voltage, and 25 °C temperature. Detection using C^4^D involved a homemade detector equipped with a sinusoidal wave generator operating at a frequency of 600 kHz and a wave amplitude of 2 Vpp. The developed CE method also facilitated the determination of additional pharmaceuticals acting as potential adulterants, such as bupropion, sertraline, paroxetine (antidepressants), and flurazepam (benzodiazepine), using identical experimental conditions. After optimization and validation, the CZE-C^4^D method was applied to detect the studied pharmaceuticals as potential adulterants in phytotherapeutic formulations marketed in Brazil as slimming products [35]. The use of CZE-C^4^D provides a reliable approach for screening multiple compounds simultaneously, enhancing the safety and quality control of such products.

The research group mentioned above further improved the developed technique by investigating potential interference from anxiolytics, diuretics, and laxatives with the detection of anorectics and antidepressants. They conducted a survey of herbal weight loss products from Brazil to assess the presence of pharmaceutical adulterants. A total of 106 herbal products, sourced from 73 pharmacies across nine Brazilian states, underwent analysis for amfepramone, bupropion, fenproporex, fluoxetine, paroxetine, sertraline, and sibutramine. This method enabled rapid and selective screening for these seven adulterants. Among the 106 weight loss products sampled, 4 (3.8%) adulterated products containing fenproporex or sibutramine were identified. These adulterated samples originated from four different pharmacies situated in three distinct Brazilian states [36]. The relatively low but significant prevalence of adulterated products highlights the ongoing challenge of ensuring the safety and efficacy of dietary supplements marketed for weight loss purposes.

Akamatsu and Mitsuhashi (2012) developed an approach employing CE for the precise quantification of glucosamine. Glucosamine, a crucial component of cartilage and widely distributed in tissues as part of glycosaminoglycans, holds significance in dietary supplements for joint health. For the extraction of glucosamine from commercial products, purified water was employed. The methodology involved in-capillary derivatization of glucosamine, with o-phthalaldehyde. CE separation was carried out using a 20 mM borate BGE at pH 9.2, supplemented with 5 mM of o-phthalaldehyde and 5 mM of 3-mercaptopropionic acid, with UV detection at 340 nm. The method exhibited an LOD of 1.3 mg/g. Subsequently, the method was applied to analyze 16 commercial products, revealing glucosamine concentrations ranging from 109 to 705 mg/g; the ratios of detected glucosamine content to the labeled values fell within the range of 88.8% to 124%. Notably, no significant bias was observed when comparing the results obtained using the proposed CE method with those obtained from an official colorimetric method [37].

Lipoic acid, a potent antioxidant, naturally exists as *R*-lipoic acid, while synthetic lipoic acid is racemic, containing both *R*- and *S*-enantiomers. Therefore, there is significant interest in determining the *R*-enantiomer content in lipoic acid supplements. Although dietary supplements may contain both racemic and *R*-lipoic acids, the exact potency of the *S*-enantiomer remains uncertain. Kodama et al. (2012) employed a chiral CE method to directly separate lipoic acid enantiomers in dietary supplements. Various factors affecting the migration time and chiral resolution of lipoic acid were explored using an OFAT approach. The optimal BGE was identified as 100 mM of phosphate buffer at pH 7.0 supplemented with 8 mM of trimethyl-β-CD as the CS. Under these conditions, successful direct chiral resolution of lipoic acid in dietary supplements was achieved. Most of the commercially available lipoic acid supplements analyzed in the study comprise the racemic mixture, although some claim to exclusively contain naturally occurring *R*-lipoic acid [38].

Moreira et al. (2013) extended their investigation to develop a method for detecting commonly used diuretics and laxatives, often found alongside anorexics and antidepressants in dietary supplement formulation, a prevalent practice in adulteration cases globally. Their CE method aimed at separating amiloride, chlorthalidone, furosemide, and hydrochlorothiazide (diuretics), along with phenolphthalein (laxative), amfepramone (anorexic), fluoxetine, and paroxetine (antidepressants), utilizing capacitively coupled with C^4^D. Among the twenty-six analyzed herbal formulations, three were identified as adulterated with the diuretic hydrochlorothiazide. Although hydrochlorothiazide typically exhibits a low incidence of side effects, its administration in high doses can result in various metabolic complications. Furthermore, five other samples contained diuretics, bringing the total to eight samples marketed as natural products but containing adulterants [39]. This study underscores the importance of robust analytical methods for identifying adulterants in herbal formulations, particularly concerning the inclusion of potentially harmful substances.

Viana et al. (2013) devised a methodology for detecting oral antidiabetic agents, including chlorpropamide, glibenclamide, gliclazide (sulphonyl urea derivatives), and metformin (biguanide derivative), using CZE coupled with C^4^D. Adulterating antidiabetic herbal preparations with hypoglycemic drugs can pose severe health risks such as hypoglycemia. Their investigation aimed to identify such adulterants in herbal formulations marketed as adjuncts in the treatment of diabetes mellitus. The proposed method demonstrated effectiveness in simultaneously identifying oral antidiabetic drugs as adulterants. It utilized a 20 mM sodium acetate solution at pH 10.0 as the BGE. Employing the C^4^D method without preconcentration techniques, the detection of organic species was achievable at concentrations as low as mg/L. This remarkable sensitivity is pivotal for both screening and quantitative analysis in cases of herbal formulation adulteration with synthetic drugs [40].

Akamatsu and Mitsuhashi (2013) introduced a CE-MS/MS method for the comprehensive analysis of free amino acids in commercial royal jelly products, which span various matrices. Royal Jelly, a creamy substance secreted by the glands of young worker honeybees, holds paramount importance in the development of queen bees and finds widespread applications in dietary supplements, medical products, and cosmetics globally. This method eliminated the need for a concentration step in sample preparation, enabling the determination of all sixteen amino acids without derivatization. The CE separation was conducted using a 1 M formic acid solution at pH 1.8 as the BGE, followed by MS/MS detection post-mixing with a sheath liquid comprising 50% (*v*/*v*) methanol. The LODs ranged from 0.61 to 10.5 μg (dry weight)/g for each amino acid. The method was applied to analyze seventeen commercial royal jelly products and compare them with honey samples. The relative proportions of free amino acids exhibited product-specific profiles, facilitating the differentiation of royal jelly products, and distinguishing them from honey. Compared to alternative techniques, this method offers notable advantages as amino acids are determined without derivatization, and sample preparation does not require concentration steps. The results underscored the characteristic levels of free amino acids present in each royal jelly product, aiding in product differentiation. Moreover, in cases where honey may be intentionally substituted for royal jelly, this method, coupled with the analysis of *trans*-10-hydroxy-2-decenoic acid, the main fatty acid in royal jelly, proves effective in uncovering such fraudulent practices [41].

Akamatsu and Mitsuhashi (2014) developed a multitarget CE-MS/MS technique for the detection of twenty pharmaceuticals commonly found in weight loss dietary supplements. These included diuretics (acetazolamide, furosemide, hydrochlorothiazide, spironolactone, triamterene, trichloromethiazide), cathartics (bisacodyl, dioctyl sulfosuccinate, picosulfate, phenolphthalein, sennoside A, B), appetite suppressants (fenfluramine, mazindol, N-didemethylsibutramine, N-nitrosofenfluramine, phentermine, sibutamine), antidepressants (fluoxetine), and anxiolytics (diazepam). This innovative method facilitated the screening and confirmation of multiple drug targets with diverse chemical structures. Separation was achieved using a BGE comprising 20 mM of ammonium formate in 20% (*v*/*v*) acetonitrile/water at pH 8 coupled with detection using a sheath liquid composed of a blend of 5 mM of ammonium formate and 0.1% *v*/*v* formic acid in 50% *v*/*v* methanol/water. The LODs ranged from 1.0 to 750 µg/g for the targeted analytes. The effectiveness of the method was demonstrated by its application to analyze twelve dietary supplements for weight loss and three non-prescription drugs. Remarkably, while ten market-purchased supplements showed no presence of pharmaceutical components, two supplements tested positive for phentermine and sibutramine, respectively [42]. This method signifies an opportunity for multi-drug target screening and confirmation using CE-MS across compounds with markedly different chemical structures.

Coelho et al. (2014) developed a CE technique for quantifying 5-hydroxytryptophan (5-HTP) in commercial dietary supplements. 5-HTP is commonly found in supplements used for various purposes, including adjuvant treatment of depression, obesity, insomnia, and chronic headaches and acting as a potent antioxidant. This amino acid is naturally synthesized from tryptophan by the enzyme tryptophan hydroxylase, and within the central nervous system (CNS), it transforms into serotonin (5-hydroxytyptamine) by tryptophan decarboxylase. Commercial 5-HTP supplements are typically derived from *Griffonia simplicifolia* seeds. The optimized BGE comprised 20 mM of phosphate at pH 10.0 and 0.2 mM of cetyltrimethylammonium bromide to invert EOF. The LOD and LOQ values for 5-HTP were determined to be 3.1 and 10.1 μmol/L, respectively. The CE method was applied to analyze four different commercial dietary supplement samples. To evaluate method accuracy, measured 5-HTP concentrations were compared to those obtained using HPLC. The results indicated no significant differences in concentrations at a confidence level of 95% [43].

Václavíková and Kvasnička (2015) addressed concerns regarding the presence of low-quality chondroitin sulfate raw materials in dietary supplement products by developing a cITP method for assessing chondroitin sulfate quality. In this method, a mixture containing 5 mM of HCl, 10 mM of glycine, and 0.01% 2-hydroxyethylcellulose at pH 2.8 served as the leading electrolyte, while the terminating electrolyte solution comprised 10 mM of citric acid. Their study included further evaluations on selected chondroitin sulfate samples, revealing that cITP, which can be used to directly analyze the chondroitin sulfate content, produced comparable results to CZE. In CZE, the chondroitin sulfate content is determined indirectly by calculating the sum of unsaturated disaccharides after treatment with chondroitinase ABC. However, it is important to note that a limitation of cITP is its inability to differentiate between dermatan sulfate and chondroitin sulfate. The need for additional enzymatic assays to differentiate between chondroitin sulfate and dermatan sulfate should be considered when implementing this method in quality control protocols [44].

dos Santos et al. (2016) developed a CE-MS/MS method for detecting amphetamine, methamphetamine, methylenedioxyamphetamine, methylenedioxymethamphetamine, methylenedioxyethylamphetamine, and phentermine in commercial samples of homeopathic and phytotherapeutic medicines, as well as dietary supplements. They employed a modified QuEChERS (Quick, Easy, Cheap, Effective, Rugged, and Safe) procedure for sample treatment, involving initial single-phase extraction followed by liquid–liquid partitioning. This method offers advantages over traditional liquid–liquid extraction (LLE), including cleaner extracts and easy isolation of the organic layer without emulsions. Electrophoretic separation was carried out in a BGE containing 100 mM of formic acid at pH 2.4 coupled with detection by ESI-MS/MS. A polyvinyl alcohol-coated capillary was utilized to prevent analyte adsorption to the capillary wall. The method achieved LOD and LOQ values ranging from 0.02 to 0.06 μg/L and 0.06 to 0.21 μg/L, respectively, with recoveries between 85% and 123%. Additionally, the separation process was completed in less than six minutes [45]. The use of CE-MS/MS coupled with a modified QuEChERS procedure offers a rapid, sensitive, and reliable method for analyzing these compounds.

**Figure 4:**
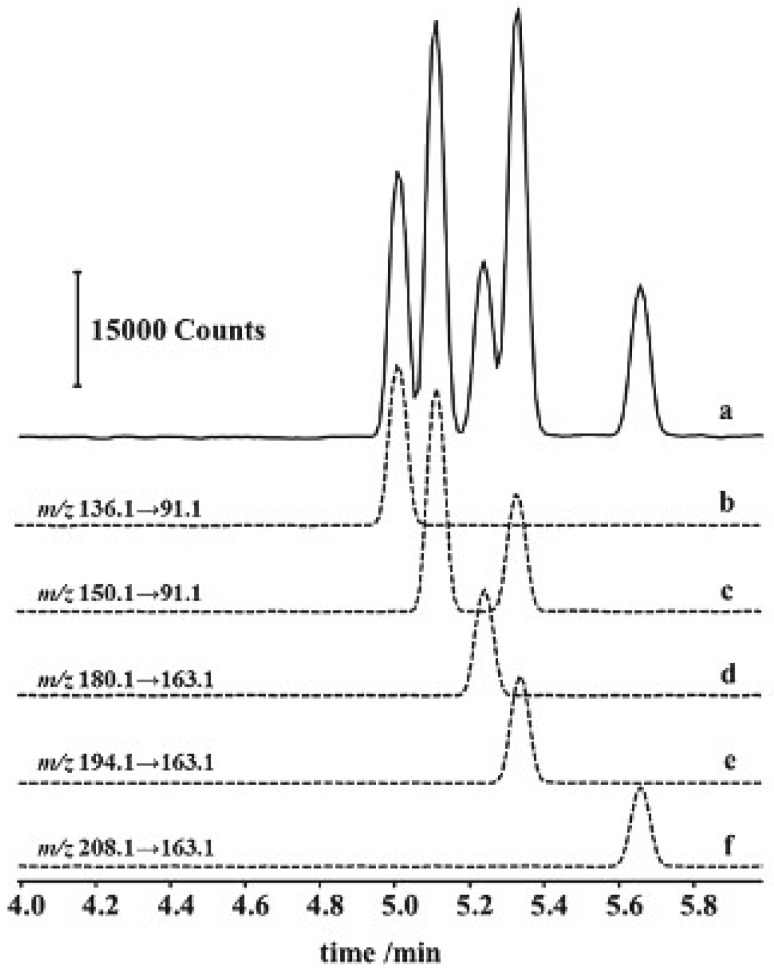
CE-MS/MS electropherogram of amphetamines and derivatives. The solid line is the total ion current (a). The dashed lines are more intense multiple reaction monitoring (MRM) transitions used for quantitative analysis of amphetamine (b), methamphetamine (first peak) and phentermine (second peak) (c), methylenedioxyamphetamine (d), methylenedioxymethamphetamine (e), and methylenedioxyethylamphetamine (f) (CE conditions: 0.1 M formic acid, pH 2.4, 25 kV, 20 °C, sheath liquid for the ESI 0.02 M formic acid, pH 2.7, prepared on methanol/water 50:50 (*v*/*v*); the flow rate was 6.0 µL/min). Reprinted from dos Santos et al. [45] with permission from Elsevier.

Figure 4 displays an electropherogram depicting a solution containing the six studied amphetamines. The order of migration in the electropherogram is contingent upon the hydrodynamic radius of the analytes, as their charge remains consistent across all species [45].

Tero Vescan et al. (2016) conducted a comparative study of weight loss dietary supplements using two separation techniques: CE and HPLC with UV detection. The study aimed to screen for caffeine, ephedrine, sibutramine, and yohimbine. For the determination of ephedrine, sibutramine, and yohimbine, a CZE method was employed, while a MEKC method was used to quantify caffeine. The findings unveiled discrepancies between the stated and actual caffeine content, attributed to the presence of both pure caffeine substance and caffeine-containing extracts within the supplement matrices. Both CE and HPLC methods yielded similar results in the analysis of dietary supplements, with any observed statistical variances likely attributable to sample uniformity [46]. By comparing CE and HPLC methodologies, the study provides valuable insights into their respective capabilities and highlights the significance of ensuring the consistency and accuracy of supplement formulations.

Wang et al. (2016) developed an MEKC technique for the simultaneous detection of sibutramine (anorexic) and phenolphthalein (laxative). Their method involved using BGE composed of a mixture of 20 mM of phosphate buffer and 20 mM of SDS at pH 11.0, with a voltage of 25 kV and a temperature of 20 °C. The influence of certain electrophoretic parameters on the separation was verified using an OFAT strategy. The evaluation of linearity, precision, and accuracy indicates that this optimized method is suitable for quantifying the concentrations of sibutramine and phenolphthalein in various slimming supplements. The LODs were determined to be 0.03 mg/mL for sibutramine and 0.18 mg/mL for phenolphthalein. Application of the proposed method demonstrated successful detection of the target chemicals in functional foods, achieving satisfactory recovery rates ranging from 95.3% to 103.4%. Furthermore, the optimized method was utilized to analyze the two target chemicals in two different brands of functional foods purchased from local drugstores, including a weight-reducing capsule sample and a weight-reducing tea sample [47].

Müller et al. (2018) conducted a CE study to detect diuretics (amiloride, chlorthalidone, furosemide, hydrochlorothiazide), antidepressants (fluoxetine, paroxetine), laxatives (phenolphthalein), and anorexics (amfepramone) in dietary supplements marketed in Brazil for weight loss and physical fitness purposes. They analyzed a total of 113 products obtained from various sources, including websites and physical stores. The analytical method employed in this study involved separating the compounds using CZE with a BGE comprising 20 mM of phosphate buffer and 30% (*v*/*v*) methanol at pH 9.2. Detection was achieved simultaneously using capacitively coupled C^4^D and UV detection. The method underwent validation following appropriate guidelines and was successfully applied to analyze the dietary supplement samples. Hydrochlorothiazide was found in fourteen of the examined samples, with one sample containing both hydrochlorothiazide and furosemide. The integration of C^4^D and UV detection systems during CZE separation of the drugs proved successful, offering valuable insights into matrix interferences. However, C^4^D appeared more susceptible to baseline oscillations due to multiple conducting and molecular species [48]. By employing CZE coupled with C^4^D and UV detection, the study provides a comprehensive approach to detecting multiple active pharmaceutical ingredients in complex supplement matrices.

Nguyen et al. (2019) developed a straightforward method for simultaneously determining taurine and choline in diverse dietary supplement and energy drink samples. Taurine and choline are commonly found in dietary supplements, particularly those aimed at enhancing physical performance, cognitive function, and overall well-being. This method utilizes dual-channeled CE instrumentation with C^4^D. The primary aim of this study was to propose a tool for food control activities, facilitating the screening of various target compounds in a single run and enabling high-throughput analysis even in settings with modest infrastructure. Taurine analysis was conducted in the first CE channel using a BGE consisting of 150 mM of tris(hydroxymethyl)aminomethane/lactic acid at pH 8.96, while choline was concurrently separated in the second CE channel using a BGE containing 150 mM of tris(hydroxymethyl)aminomethane/acetic acid at pH 9.5. The method achieved LODs of 0.27 mg/L for taurine and 0.45 mg/L for choline. The results obtained from CE-C^4^D showed good agreement with those obtained using standard confirmation methods (HPLC-DAD for taurine and LC-MS for choline). This study underscores the utility of CE-C^4^D as a straightforward and economical solution for rapid screening of various food additives in different food matrices. Additionally, the use of independent CE conditions in different channels adds a positive feature to CE-C^4^D, allowing for the concomitant determination of various analyte categories with different chemical characteristics [49].

Duong et al. (2020) developed an approach for quantifying 10-hydroxy-2-decenoic acid and free amino acids in various royal-jelly-based dietary supplements, using a custom-built dual-channeled CE system paired with C^4^D detection. *Trans*-10-hydroxy-2-decenoic acid serves as a crucial marker for assessing the quality of royal jelly as it plays a pivotal role in enhancing immune function and exhibiting antibacterial properties. Throughout the product’s shelf life, royal jelly is susceptible to alterations that affect its amino acid composition, potentially leading to browning during storage at room temperature; consequently, 10-hydroxy-2-decenoic acid, along with other free amino acids, is commonly utilized as an indicator to evaluate the quality of royal jelly. For 10-hydroxy-2-decenoic acid, the BGE consisted of 20mM Tris(hydroxymethyl)aminomethane adjusted to pH 8.5 with acetic acid, while for free amino acids, it comprised 2M of lactic acid. The developed CE-C^4^D methods achieved LODs of 0.039 mg/g for 10-hydroxy-2-decenoic acid and a range of 0.039–0.090 mg/g for free amino acids. The study demonstrated strong agreement between the results obtained via CE-C^4^D and those from standard confirmation methods like HPLC-PDA; the deviations between the two sets of data are less than 5% for 10-hydroxy-2-decenoic acid and 16% for free amino acids. This reinforces the reliability and accuracy of CE for analyzing these compounds in royal-jelly-based dietary supplements [50].

Restaino et al. (2020) introduced a CE method for quantifying chondroitin sulfate, keratan sulfate, and hyaluronic acid as intact chains. Chondroitin sulfate, derived from animal cartilage, is widely used as an active agent against osteoarthritis, but its efficacy can be affected by impurities like keratan sulfate or intentionally added substances like hyaluronic acid in food supplements. In this research, the CE method was applied to analyze five chondroitin sulfate standards and thirteen samples from various animal sources or food supplement suppliers. Notably, this method not only accurately quantified keratan sulfate, comparable to a previously reported high-performance anion-exchange chromatography method, but also identified the presence of hyaluronic acid, a capability not previously reported. The CE method’s precision and accuracy were similar to techniques based on monosaccharide and disaccharide analyses but offered the advantage of bypassing hydrolytic pre-treatments or labeling reactions typically required [51].

Kvasnička and Rajchl (2021) devised an electrophoretic method combining cITP and CZE with conductometric detection for the determination of free taurine in selected food and feed samples. Taurine is initially converted to isethionic acid using the van Slyke method. Optimized conditions involved a leading electrolyte composed of 5 mM of HCl, 10 mM of glycylglycine, and a 0.05% solution of 2-hydroxyethyl cellulose at pH 3.2, a terminating electrolyte of 10 mM of citric acid, and a BGE containing 50 mM of acetic acid, 20 mM of glycylglycine, and a 0.1% solution of 2-hydroxyethyl cellulose at pH 3.7. Within 15 min, isethionic acid was separated from other sample components in anionic mode and detected using a conductometer. The method’s LOD and LOQ were determined to be 3 ng/mL and 10 ng/mL, respectively. Analysis of food and pet food samples confirmed the method’s suitability for routine analysis. The method’s advantages include a short analysis time (15 min), high selectivity and sensitivity (~ng/mL), and low cost (requiring only a small volume of aqueous diluted electrolyte) [52]. The combination of cITP and CZE with conductometric detection enhances selectivity and sensitivity, making it suitable for routine analysis in laboratory settings.

Riasova et al. (2022) developed and validated a CE method for the baseline separation of closely related flavonolignans: silybin A, silybin B, isosilybin A, isosilybin B, silychristin, silydianin, and their precursor taxifolin in silymarin complex. Silymarin, derived from milk thistle (*Silybum marianum*), has traditionally been used in treating liver disorders like hepatitis, cirrhosis, and toxin-induced liver dysfunction. The optimized BGE consisted of 100 of mM boric acid at pH 9.0 supplemented with 10% (*v*/*v*) methanol and 5 mM of heptakis(2,3,6-tri-O-methyl)-β-CD. Genistein served as an IS. The method was applied to determine flavonolignans in two dietary supplements containing *Silybum marianum* extract. Accuracy was assessed by comparing CE analysis results with those from the reference United States Pharmacopeia (USP) HPLC method. Statistical analysis via an unpaired t-test revealed no significant difference between the proposed CE and the HPLC reference method. Despite the CE method’s approximately 25-minute runtime, it remained quicker than USP HPLC or European Pharmacopoeia (Ph. Eur); these methods exceed 50 min without achieving baseline separation of all six flavonolignans. The findings validated that the two examined supplements met the criteria outlined by the USP and Ph. Eur. for milk thistle dried extract. Additionally, the CE method was deemed to be more environmentally friendly compared to LC methods [53].

**Figure 5:**
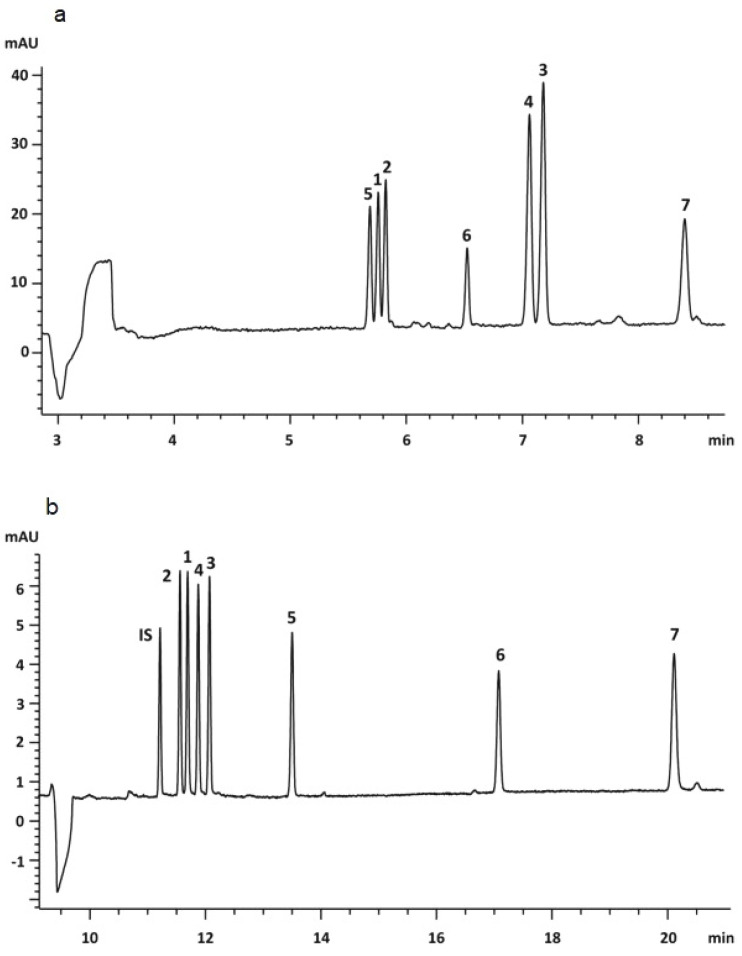
Electropherograms of flavonolignans (1—silybin A, 2—silybin B, 3—isosilybin A, 4—isosilybin B, 5—silychristin, 6—silydianin, 7—taxifolin, IS—genistein) (CE conditions: method (**a**) MEKC: 100 mM of borate, 140 mM of SDS, 10% methanol, 5 mM of hydroxypropyl-β-CD, pH 9.0, 25 kV, 25 °C; method (**b**)—EKC: 100 mM of borate, 10% methanol, 5 mM of heptakis(2,3,6-tri-O-methyl)-β-CD, pH 9.0, 25 kV, 25 °C, UV detection 200 nm). Reprinted from dos Riasova et al. [53] with permission from Wiley.

Figure 5 shows EKC and MEKC electropherograms of flavonolignans [53].

Cizmarova et al. (2022) developed an innovative two-dimensional CE method, integrating cITP and CZE separation steps, coupled with highly sensitive and MS detection, for quantifying two B vitamins, namely thiamine (vitamin B1) and pyridoxine (vitamin B6), across various pharmaceutical samples and dietary supplements. The analytical method demonstrated ultralow detection limits in the picogram per milliliter (pg/mL) range. This method was effectively validated and successfully applied in the quality control processes of three pharmaceutical formulations containing thiamine and pyridoxine, including tablets, effervescent tablets, and drops. The results obtained from the Analytical GREEnness metric approach and the semi-quantitative Analytical Eco-Scale approach indicated that the method serves as an eco-friendly approach for the targeted and highly sensitive quantification of the selected B vitamins [54]. The eco-friendly nature of the method aligns with current trends in analytical chemistry, emphasizing sustainability and environmental consciousness in laboratory practices.

Amorim et al. (2020) developed a CE method to determine docosahexaenoic acid and eicosapentaenoic acid in marine oil Omega-3 supplements. These essential Omega-3 fatty acids are vital for human health and must be obtained through diet, as the body cannot synthesize them. Omega-3 supplements, derived from fish and/or krill oils, are available in various formulations and often claim to be “high in omega-3 fatty acids” to attract consumers, highlighting their potential to help prevent coronary heart disease. The CE method involved a simple saponification step and had an analysis time of 8 min. When tested on ten real samples, the results from the CE method were consistent with those obtained using GC, with no significant differences observed within a 95% confidence interval [55].

**Figure 6:**
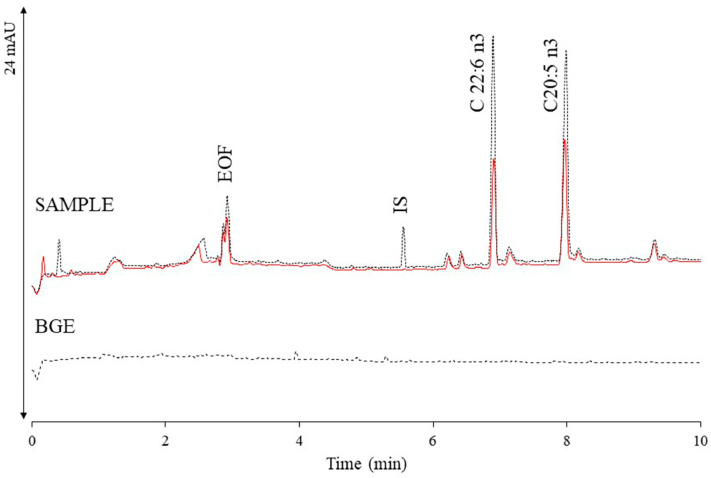
BGE signal and electropherograms of a fish oil sample injected alone (solid line) and spiked with a mixture of elaidic acid (C18:1 trans-9) (IS), eicosapentaenoic acid (C20:5 n3) and docosahexaenoic acid (C22:6 n3) (dotted line) (CE conditions: 30 mM of sodium tetraborate, 12 mM of Brij 35, 33% methanol (*v*/*v*), 17% acetonitrile (*v*/*v*), 27 kV, 27 °C, UV detection 200 nm). Reprinted from Amorim et al. [55] with permission from Elsevier.

Figure 6 presents the electropherograms under optimized CZE-UV conditions, comparing the BGE signal, a marine oil sample (solid line), and the same sample spiked with elaidic acid (IS), eicosapentaenoic acid, and docosahexaenoic acid (dotted line).

Do et al. (2023) developed a CE-C^4^D method to simultaneously determine glucosamine and calcium (Ca^2+^) in dietary supplements. Using optimized conditions, the method showed excellent linearity for glucosamine and Ca^2+^, with LODs of 1 mg/L and 0.05 mg/L, respectively. The total analysis time was approximately 5 min. The validated method successfully analyzed seven dietary supplement samples, with results aligning with label claims and with results of reference methods, HPLC with a fluorescence detector (FLD) for glucosamine and inductively coupled plasma optical emission spectrometry (ICP-OES) for Ca^2+^ [56].

Pukleš et al. (2023) introduced a new microchip electrophoresis with capacitively coupled contactless conductivity detection (MCE-C^4^D) method for the electrophoretic determination of *L*-carnosine in dietary supplements. The method does not require pre-concentration or derivatization steps. Validation against CE-UV-VIS and HPLC-DAD confirmed the method’s accuracy [57]. The same research group developed another microfluidic method for determining *L*-histidine and β-alanine in dietary supplements using the same MCE-C^4^D technique. The method allows the identification of amino acids without chemical derivatization and can simultaneously analyze amino acids and metal cations. LOD values for *L*-histidine and β-alanine were 4.2 µmol/L and 5.2 µmol/L, respectively. Accuracy was confirmed through recovery experiments and comparison with CE-UV-VIS and HPLC-UV-VIS techniques. The microfluidic method was successfully applied to three commercial dietary supplements, showing results consistent with product labels [58]. The key advantages of MCE-C^4^D include simplicity, speed, efficiency, low cost, portability, and reduced environmental impact. The study highlights the potential for future research to develop single-run methods for the simultaneous analysis of diverse chemical species in complex formulations.

A summary of studies focusing on the adulteration control of dietary supplements utilizing CE methodologies published in the last two decades is presented in Table 1.

## 4. Challenges and Considerations

In both the EU and USA, dietary supplements are not required to undergo specific regulatory preapproval or safety assessments before they are marketed. This regulatory gap provides an opportunity for unscrupulous manufacturers and distributors to deliberately adulterate supplements by adding approved pharmaceutical drugs or analog substances that have not been fully evaluated for their effectiveness or toxicity. These practices are typically used to boost the perceived effectiveness of the product and increase sales, which can pose serious risks to consumer health and safety [2,4,5].

Over the past two decades, researchers have demonstrated the utility of CE methods in the analysis of adulterated dietary supplements, including CZE, MEKC, and cITP, alongside various detection techniques such as UV, MS, and C^4^D. These efforts have been directed towards addressing persistent challenges associated with detecting potential adulterants, quantifying active compounds, and screening for impurities in dietary supplements and herbal products [8,20,59].

One focal point of these studies has been the detection of synthetic compounds often found in adulterated products, including PDE-5 inhibitors, anorectic agents, antidepressants, diuretics, laxatives, or antidiabetic drugs. Achieving sensitive and reliable detection of these compounds required meticulous optimization of CE conditions, including the composition and pH of BGE, use of BGE additives, voltage, and temperature.

Additionally, CE methods have been developed for the chiral separation of bioactive compounds such as ephedrine alkaloids and their enantiomers, commonly present in herbal supplements. Utilizing CDs as CSs, along with the optimization of BGE composition and addition of organic modifiers, facilitates efficient separation and quantification.

CE techniques have been applied to quantify natural compounds like catechins in green tea extracts or *L*-carnitine in dietary supplements, offering rapid and reliable analysis methods. This enabled the assessment of product quality and authenticity by comparing measured concentrations with labeled values. Furthermore, CE methods have been instrumental in detecting impurities and adulterants, ranging from glycosaminoglycans in chondroitin sulfate products to synthetic compounds in weight loss supplements. By enabling simultaneous screening of multiple compounds, these methods ensure compliance with regulatory standards and safeguard consumer health.

The optimization of separation conditions, sample preparation techniques, and detection methods underscores a dedicated effort to improve the accuracy, reliability, and efficiency of product analysis. Comparison with conventional techniques like HPLC highlights the respective advantages and limitations, aiding researchers in selecting the most suitable method for their study [60].

While CE has shown significant advantages, it often complements traditional methods like HPLC and GC. CE’s rapid analysis time, low sample and reagent consumption, and relatively low operational costs make it a valuable tool for routine quality control and adulteration detection. However, careful method development and validation are essential to address potential matrix interferences and ensure comprehensive analyte profiling [20,60,61].

## 5. Review Methodology

A comprehensive literature search was conducted to identify relevant studies for inclusion in this review. Electronic databases including Google Scholar, Scopus, and Web of Science were searched using relevant keywords: “dietary supplements”, “capillary electrophoresis”, and “adulterants”. Boolean operators “and” and “or” were used to combine search terms appropriately. Additionally, reference lists of relevant articles were manually screened to identify additional studies missed during the initial search. Data synthesis was performed using a narrative approach, summarizing key findings and themes across the included studies.

## 6. Conclusions

With the increasing consumption of dietary supplements and the expansion of the global market, stronger oversight by regulatory authorities has become essential. Detecting adulterations and taking enforcement actions when necessary are critical steps to protect public health. Consequently, the development of advanced analytical methods to identify adulterants, including new or unknown analogs from various pharmacological classes, is challenging and crucial.

CE has become a powerful tool for analyzing natural products, including dietary supplements and herbal products, particularly in detecting adulterants. Over the past two decades, techniques like CZE, MEKC, cITP, and CE-MS have shown high sensitivity, specificity, and efficiency in analyzing various compounds.

Applying CE to detect adulterants requires careful method optimization due to the complex nature of supplements. Key factors include careful sample preparation, including extraction and filtration, as well as the selection of the appropriate separation mode (CZE or MEKC) based on the characteristics of the adulterants and the supplement matrix. Optimizing BGE composition, pH, voltage, and temperature, along with coupling CE with appropriate detection methods like UV, fluorescence, or MS, ensures accurate and reliable results, complementing traditional methods like HPLC and GC.

Recent advancements in CE, including MEKC and improved CE-MS coupling, have further enhanced its effectiveness in detecting adulterants. Innovations like CE-MS/MS are particularly promising for analyzing complex matrices and detecting low-concentration adulterants. Microchip electrophoresis (MCE) offers additional benefits, such as increased speed, efficiency, and portability for simultaneous detection of multiple adulterants. Future research should focus on developing sustainable, environmentally friendly CE methods, in line with green analytical chemistry principles.

While fewer CE studies focus on dietary supplement quality control compared to HPLC, CE holds considerable potential. CE offers advantages such as rapid method development, low sample and reagent usage, and versatility in detecting various analytes. However, challenges include matrix interference from complex supplement compositions, which can affect accuracy and sensitivity, and limitations in separating certain compounds, potentially impacting comprehensive adulterant detection.

Looking ahead, continued efforts in method development will be essential to further enhance sensitivity, selectivity, and throughput in CE analysis. Additionally, expanding the scope of analysis to encompass a broader range of compounds and product types will be crucial for addressing evolving challenges in product adulteration.

## Figures and Tables

**Table 1:** Studies on adulteration control of dietary supplements using CE methodologies.

CE Technique	Analytical Conditions	Product	Substances	Reference
CZE	120 mM of phosphate, 15% acetonitrile, pH 2.0, 16 kV, 30 °C UV detection 200 nm	traditional Chinese medicinal powders	clobenzorex, diethylpropion, fenfluramine, methamphetamine, phenylpropanolamine, phentermine	[21]
CZE MEKC CE-ESI-MS	CZE: 40 mM of ammonium acetate, pH 9.0, 20 kV MEKC: 40 mM of ammonium acetate, 20 mM of SDS, pH 9.0, 20 kV UV detection 254 nm CE-ESI-MS: sheath liquid composition methanol/water (70:30) containing 0.2% formic acid, ESI voltage +4 kV	traditional Chinese medicinal powders	acetaminophen, bucetin, caffeine, diazepam, ethoxybenzamide, fenbufen, flufenamic acid, indomethacin, ketoprofen, mefenamic acid, niflumic acid, oxyphenbutazone, phenylbutazone, prednisolone, salicylamide, sulindac	[23]
CZE	100 mM of phosphate–borate, pH 7.50, 15 kV, 30 °C UV detection 200 nm	traditional Chinese medicines	acetohexamide, chlorpropamide, glibenclamide, tolbutamide	[24]
CE	55 mM of phosphate, 10 mM of borate, 20 mM of HP-β-CD, 7 mM of β-CD, 30% methanol, pH 8.6, 30 kV, 37 °C UV detection 210 nm	tablets, capsules	ephedrine, pseudoephedrine stereoisomers	[25]
CZE	50 mM of phosphate, 20% methanol, pH 7.0, 15 kV, 25 °C UV detection 208 nm	tablets	alpha-lipoic acid	[26]
CZE	20 mm of sodium tetraborate, pH 9.2, 25 kV, 25 °C UV detection 212 nm	guarana powder and tablets	caffeine, theobromine, theophylline	[27]
CZE	Method I: 25 mM of phosphate, pH 2.5, 2.8% sulfated-β-CD + 1.2% heptakis(2,6-di-O-methyl)-β-CD, −15 kV, 25 °C Method II: 25 mM of phosphate, pH 2.5, 4% 4% heptakis(2,6-di-O-methyl)-β-CD, 30 kV, 25 °C Method III: 25 mM of phosphate, pH 2.5, 4% hydroxypropyl-β-CD, 25 kV, 25 °C UV detection 210 nm	ephedra products	ephedrine, pseudoephedrine stereoisomers	[28]
cITP CZE	cITP: terminating electrolyte 10 mM of acetic acid, leading electrolyte 40 mM of acetic acid, 20 mM of ammonium hydroxide CZE: 15 mM of Tris, 25 mM of phosphate, 15 kV UV detection 254 nm	food supplements	*L*-carnitine	[29]
MEKC	5 mM of borate, 60 mM of phosphate, 50 mM of SDS, pH 7.00, 27 kV, 25 °C UV detection 210 nm	dietary supplements containing green tea extracts	caffeine, catechins ((−)-epigallocatechin, (+)-catechin, (−)-epigallocatechin-3-gallate, (−)-epicatechin, or (−)-epicatechin gallate)	[30]
CZE	50 mM of phosphate, pH 3.0, 30 kV, 25 °C UV detection 200 nm	tablets, hard capsules, liquid formulations	chondroitin sulfate (glycosaminoglycan or DNA impurities, hyaluronan impurities)	[32]
CZE	23 mM of borate, 7% acetonitrile (*v*/*v*), pH 10.0, 26 kV, 20 °C UV detection 280 nm	effervescent tablets	resveratrol	[33]
CE-ESI-MS	50 mM of ammonium formate, pH 2.5, 0.2% (*m*/*v*) succinyl-γ-CD (4 succinyl groups/CD ring), 25 kV, 25 °C MS: sheath liquid, isopropanol/water (50/50 *v*/*v*) with 0.1% formic acid	tablets, capsules, biscuits, drinks	carnitine enantiomers	[34]
CZE	50 mM of phosphate in a mixture of water/acetonitrile 50/50 (*v*/*v*), 15 kV, 25 °C C^4^D detection at 600 kHz and 2 Vpp	slimming capsules	amfepramone, fenproporex, fluoxetine, sibutramine	[35]
CZE	20 mM of borate, 5 mM of o-phthalaldehyde (derivatization agent), 5 mM of 3-mercaptopropionic acid, pH 9.20, 25 kV, 30 °C UV detection 340 nm	dietary supplements	glucosamine	[36]
CE	100 mM of phosphate, pH 7.0, 8 mM of TM-β-CD, 18 kV, 20 °C UV detection 200 nm	dietary supplements	alpha-lipoic acid (chiral purity)	[38]
CZE	20 mM of phosphate + 30% methanol, pH 9.2, 15 kV, 25 °C C^4^D detection at 400 kHz and 2 Vpp	slimming capsules	amiloride, chlorthalidone, furosemide, hydrochlorothiazide phenolphthalein amfepramone fluoxetine, paroxetine	[39]
CZE	20 mM of sodium acetate, pH 10.0, −15 kV, 25 °C C^4^D detection at 400 kHz and 2 Vpp	antidiabetic capsules	chlorpropamide, glibenclamide, gliclazide, metformin	[40]
CE-MS-MS	20 mM of ammonium formate in 20% acetonitrile, pH 8.0, 30 kV MS: sheath liquid, 5 mM of ammonium formate and 0.1% (*v*/*v*) formic acid in 50% *v*/*v* methanol/water	slimming tablets, capsules, powders	acetazolamide, furosemide, hydrochlorothiazide, spironolactone, triamterene, trichloromethiazide bisacodyl, dioctyl sulfosuccinate, picosulfate, phenolphthalein, sennoside A, B fenfluramine, mazindol, N-didemethylsibutramine, N-nitrosofenfluramine, phentermine, sibutamine fluoxetine diazepam	[42]
CZE	20 mM of phosphate, pH 10.0, 0.2 mM of CTAB, −20 kV, 25 °C UV detection 214 nm	tablets and capsules	5-hydroxytryptophan (5-HTP)	[43]
cITP CZE	cITP: leading electrolyte: 5 mM of hydrochloric acid + 10 mM of glycine + 0.01% of 2-hydroxyethylcellulose, pH 2.8; terminating electrolyte: 10 mM of citric acid UV detection 254 nm CZE: 25 mM of phosphate + 21 mM of Tris, pH 3.0, −20 kV UV detection 232 nm	raw material used for dietary supplements production	chondroitin sulphate	[44]
CE-MS-MS	100 mM of formic acid, pH 2.4, 25 kV, 20 °C MS: sheath liquid 20 mM of formic acid, pH 2.7, in methanol/water 50:50 (*v*/*v*)	tablets, capsules, tea	amphetamine, methamphetamine, methylenedioxyamphetamine, methylenedioxymethamphetamine, methylenedioxyethylamphetamine and phentermine	[45]
CZE MEKC	50 mM of phosphate, pH 4.8, 25 kV, 20 °C 50 mM of sodium tetraborate + 50 mM of SDS, pH 9.8, 25 kV, 20 °C UV detection 210 nm	slimming tablets, capsules	caffeine, ephedrine, sibutramine, yohimbine	[46]
MEKC	20 mM of phosphate + 20 mM of SDS, pH 11.0, 25 kV, 20 °C UV detection 223 nm	slimming capsules, tea	phenolphthalein, sibutramine	[47]
CZE	20 mM of phosphate, 40 mM of sodium hydroxide + 30% methanol (*v*/*v*), pH 9.2, 15 kV, 25 °C UV detection 260 nm C^4^D detection at 400 kHz	weight loss, fat burning, appetite reduction supplements	amiloride, chlorthalidone, furosemide, hydrochlorothiazide fluoxetine, paroxetine phenolphthalein amfepramone	[48]
CZE	choline: 150 mM of Tris/lactic acid, pH 8.96, −10 kV taurine: 150 mM of Tris/acetic acid, pH 9.5, −10 kV C^4^D detection at 400 kHz	energy drinks, powdered infant formula, probiotic green rice samples	choline, taurine	[49]
CE	10-hydroxy-2-decenoic acid: 20 mM of Tris(hydroxymethyl)aminomethane, pH8.5, −17 kV free amino acids: 2 M lactic acid C^4^D detection 400 kHz	royal jelly based dietary supplement	10-hydroxy-2-decenoic acid, free amino acids	[50]
cITP-CZE	leading electrolyte: 5 mM of HCl, 10 mM of glycylglycine 0.05% 2-hydroxyethyl cellulose solution, pH 3.2 terminating electrolyte: 10 mM of citric acid BGE electrolyte: 50 mM of acetic acid, 20 mM of glycylglycine, and 0.1% 2-hydroxyethyl cellulose solution, pH 3.7	dietary supplements, food samples	taurine	[52]
CZE	100 mM of boric acid, 10% methanol, 5 mM of heptakis(2,3,6-tri-O-methyl)-β-CD, pH 9.0, 25 kV, 25 °C UV detection 200 nm	dietary supplements	flavonolignans (silybin A, silybin B, isosilybin A, isosilybin B, silychristin, silydianin) in silymarin	[53]
cITP-CE-ESI-MS	10 mM of ammonium acetate + 20 mM of acetic acid, pH 4.5 spray liquid mixture of methanol + 0.1% acetic acid water solution (50:50, *v*/*v*)	tablets, effervescent tablets, drops	pyridoxine (vitamin B6), thiamine (vitamin B1)	[54]
CZE	30 mM of sodium tetraborate, 12 mM of Brij 35, 33% methanol (*v*/*v*), 17% acetonitrile (*v*/*v*), 27 kV, 27 °C, UV detection 200 nm	marine oil gelulels	eicosapentaenoic acid, docosahexaenoic acid	[55]
CZE	10 mM of Tris/acetic acid, pH 5.0, 20 kV, 20 °C C^4^D detection at 400 kHz	tablets, hard capsules	glucosamine, calcium	[56]

## Abbreviations

BGE—background electrolyte; CD—cyclodextrin; C4D—contactless conductivity detection; CE—capillary electrophoresis; CNS—central nervous system; CS—chiral selector; CZE—capillary zone electrophoresis; cITP—capillary isotachophoresis; DAD—diode array detector; DoE—design of experiment; EKC—electrokinetic chromatography; EMA—European Medicines Agency; EOF—electroosmotic flow; ESI—electrospray ionization; FDA—Food and Drug Administration; GC—gas chromatography; 5-HTP—5-hydroxytryptophan; HPLC—high-performance liquid chromatography; ICH—International Conference on Harmonization; IS—internal standard; LC—liquid chromatography; LLE—liquid–liquid extraction; LLOQ—lower limit of quantitation; LOD—limit of detection, LOQ—limit of quantification; MCP—microchip capillary electrophoresis; MEKC—micellar electrokinetic chromatography; MS—mass spectrometry; NMR—nuclear magnetic resonance; OFAT—one factor at a time; PDA—photodiode array; SDS—sodium dodecyl sulfate; SPE—solid phase extraction; SSRI—selective serotonin reuptake inhibitor; TLC—thin-layer chromatography; UV—ultraviolet
